# Endoscopic Ultrasound Quality Metrics in Clinical Practice

**DOI:** 10.3390/diagnostics11020242

**Published:** 2021-02-04

**Authors:** Lawrence Ku, Linda A. Hou, Viktor E. Eysselein, Sofiya Reicher

**Affiliations:** Division of Gastroenterology, Harbor-UCLA Medical Center, Torrance, CA 90502, USA; lawrenceku@gmail.com (L.K.); lindahou@gmail.com (L.A.H.); veysselein@lundquist.org (V.E.E.)

**Keywords:** endoscopic ultrasonography, quality indicators, healthcare, fine needle biopsy

## Abstract

Recent advances in endoscopic ultrasound (EUS), particularly EUS-guided tissue acquisition, may have affected EUS procedural performance as measured by current American Society for Gastrointestinal Endoscopy (ASGE)/American College of Gastroenterology (ACG) quality indicators. Our study aims to assess how these quality metrics are met in clinical practice. We retrospectively analyzed 732 EUS procedures; data collected were procedural indications, technical aspects and outcomes, completeness of documentation, and malignancy staging. EUS was performed in 660 patients for a variety of indications. All ASGE/ACG EUS procedural quality metrics were met or exceeded. Intervention was successful in 97.7% (715/732) of cases, with complication rate of 0.4% (3/732). EUS outcomes changed clinical management in 58.7% of all cases and in 91.2% of malignancy work-up cases; in 26.0% of suspected choledocholithiasis cases, endoscopic retrograde cholangiopancreatography (ERCP) was avoided after EUS. Locoregional EUS staging was accurate in 61/65 (93.8%) cases of non-metastatic disease and in 15/22 (68.2%) cases of metastatic disease. Pancreatic mass malignancy detection rate with EUS-guided fine needle aspiration (FNA) or fine needle biopsy (FNB) was 75.8%, with a sensitivity of 96.2%; a significant increase in detection rate from 46.2% (6/13) to 95.0% (19/20) (*p* = 0.0026) was observed with a transition to the predominant use of FNB for tissue acquisition. All ASGE/ACG EUS quality metrics were met or exceeded for EUS procedures performed for a wide variety of indications in a diverse patient population. EUS was instrumental in changing clinical management, with a low complication rate. The malignancy detection rate in pancreatic masses significantly increased with FNB use.

## 1. Introduction

Endoscopic ultrasound (EUS) is an indispensable modality for diagnosis of various gastrointestinal (GI) pathologies [1,2,3]. Widespread applications of EUS, such as fine needle aspiration (FNA), are well studied and have been incorporated into practice guidelines, whereas more recent applications, such as fine needle biopsy (FNB), are comparatively less studied. To ensure judicious use of EUS, the American Society for Gastrointestinal Endoscopy (ASGE) and the American College of Gastroenterology (ACG) proposed a set of quality indicators for EUS in 2006, with a subsequent revision in 2015 [4]. In addition, the ASGE published an updated list of appropriate indications for EUS in 2012 [5]. Based on clinical significance, priority has been given to three specific indicators: appropriate GI cancer staging, diagnostic rates of malignancy and sensitivity for EUS-guided FNA (EUS-FNA) of pancreatic masses, and incidence of adverse events after EUS-FNA. Specifically, the metrics have been (1) >80% frequency for which EUS was performed for a standard indication; (2) ≥85% diagnostic rate of adequate sample for all solid lesions undergoing EUS-FNA; (3) ≥70% diagnostic rate and ≥85% sensitivity for malignancy in EUS-FNA of pancreatic masses; (4) <2% incidence of acute pancreatitis, <0.5% incidence of perforation, and <1% incidence of clinically significant bleeding in patients who undergo EUS-FNA. The guidelines also recommended that >98% of GI cancers are staged with the American Joint Committee on Cancer (AJCC) staging system.

Evaluations of pancreatic masses and benign biliary diseases are two main indications for EUS. Recent ASGE guidelines on the management of choledocholithiasis recognized that earlier (2010) guidelines for proceeding directly to endoscopic retrograde cholangiopancreatography (ERCP) for patients stratified to a high probability of choledocholithiasis were neither sensitive nor specific enough; thus, the clinical management algorithm has been updated in 2019 to incorporate modalities such as EUS [6,7].

The ASGE/ACG quality metrics for EUS were graded based on the availability and quality of supporting evidence at the time. Recent advances in EUS, such as FNB, call for an assessment of EUS performance in clinical practice in relation to the current quality indicators. There are limited data on the performance of these indicators for broad EUS outcomes, especially in a diverse patient population [8,9]. Here, we evaluate how our EUS practice adheres to and meets the ASGE/ACG quality metrics, and whether EUS has been instrumental in clinical management.

## 2. Materials and Methods

We retrospectively collected data on all patients who underwent EUS from 1 July 2016 to 31 January 2018 at our institution, a large tertiary safety net hospital. Data were collected in two time periods: July 2016 through March 2017, and April 2017 through January 2018.

Procedures were performed by three experienced endosonographers who each have performed over 1000 EUS procedures. The choice of sedation method for procedures was left to the discretion of the anesthesiologist; in most cases, intravenous sedation with propofol was used.

The echoendoscopes used were GF-UE160-AL5, GF-UC140-AL5, GF-UC140P-AL5, and GF-UCT180 (Olympus America, Center Valley, PA, USA). EUS-FNA and FNB needles were from a variety of manufacturers: Expect FNA and Acquire FNB needles (Boston Scientific, Natick, MA, USA), SharkCore FNB needles (Medtronic, Dublin, Ireland), and Echo Tip ProCore FNB needles (Cook Medical, Bloomington, IN, USA). Linear and radial echoendoscopes were used for the staging of malignancy. For FNA and FNB of lesions, we used a fanning technique, with 10 actuations per lesion.

Data collected from hospital electronic health records and EUS databases included patient demographics, comorbidities, laboratory studies, clinical outcomes, pathology, and locoregional and final malignancy staging when indicated. Procedure-related data included indications, technical aspects, complications, and accuracy and completeness of documentation.

Tumors were staged following the AJCC 8th Edition Cancer Staging Manual [10].

Emergent procedures were those performed in the inpatient setting within 12 h of admission; urgent procedures were all others performed in the inpatient setting; outpatient procedures were considered elective.

Change in management (as documented in the electronic health record) was defined as follows: avoidance of ERCP if EUS showed no choledocholithiasis; staging of malignancy leading to changes in multidisciplinary plan of care; and confirmation or rejection of a suspected diagnosis leading to changes in the plan of care.

Statistical analysis was performed using Fisher’s exact test, with *p* value < 0.05 as statistically significant; all analyses were performed with R version 3.6.0 (R Core Team, Vienna, Austria). The study was approved on 20 September 2017 by the Institutional Review Board (#31297-01).

## 3. Results

### 3.1. Demographics and Procedure Metrics

Over a 19-month period, 732 consecutive EUS procedures were performed in 660 patients (Table 1). The average patient age was 51.0 years (15–92), and 38.8% were males. EUS was successfully performed in 97.7% of cases. ERCP was performed in the same session 46.7% of the time. FNA or FNB was performed in 106 (14.5%) cases, with an average of 3.3 passes per needle.

Compared to patients undergoing evaluation for choledocholithiasis, those undergoing evaluation for malignancy were older (47.1 years vs. 57.3 years). Both groups had a female predominance (male 30.6% vs. 42.1%).

Of documented procedure indications, 95.8% (701/732) met ASGE guidelines (Table 2). All patients undergoing EUS-FNA of cysts received prophylactic antibiotics. One hundred percent of cases had documentation of sedation-related events, key anatomy and landmarks, and plans for patient follow-up. Photo or video documentation was present in 97.5% of cases; all missing cases were due to equipment malfunction.

### 3.2. Indications

The prevalent indications for EUS (Table 3) were choledocholithiasis in 44.7% (327/732) and malignancy work-up in 37.3% of cases (273/732). The most common among the 273 malignancy cases were gastric 24.2% (66/273), pancreatic mass 22.3% (61/273), and rectal 17.6% (48/273); other malignancies included esophageal, mediastinal, biliary, and small bowel. In addition to choledocholithiasis, other benign conditions evaluated included biliary and liver disease, pancreatic cyst, pancreatitis, and rectal incontinence.

Overall, 95.8% (701/732) of procedures met an indication described in ASGE guidelines [5]. Of the 31 cases that did not meet the guidelines, one was for unexplained abdominal pain, which was not fully documented in the EUS report; the other thirty were in patients with high-risk predictors for choledocholithiasis (total bilirubin > 4.0 mg/dL) for which EUS was not recommended in the 2010 ASGE guidelines [6].

### 3.3. Outcomes: General

**Figure 1:**
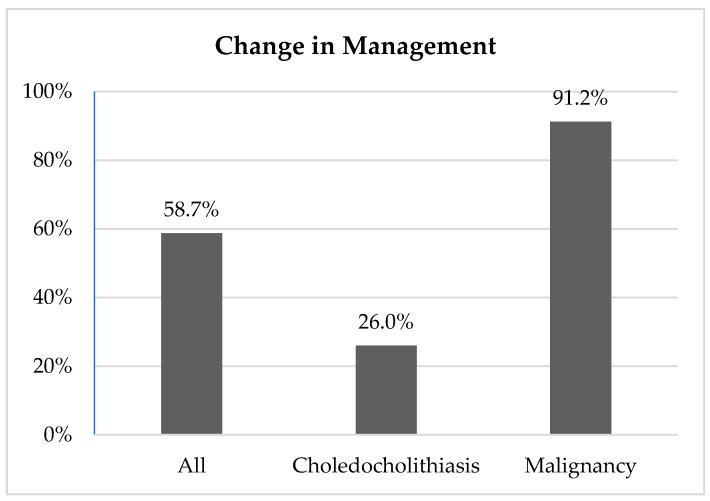
Change in management.

Overall, EUS changed clinical management in 58.7% (430/732) of cases. The management plan was changed in 26.0% (85/327) of choledocholithiasis cases; in 91.2% (249/273) of malignancy work-up cases; and in 72.7% (96/132) of other benign conditions such as pancreatic, liver, and biliary disease (Figure 1).

### 3.4. Outcomes: Malignancy

EUS was performed for locoregional staging of esophageal, gastric, pancreatic, and rectal malignancies. There were 91 instances of malignancy staged by EUS. The median tumor stage by EUS was T3. Final staging revealed metastatic disease in 22/91 (24.2%) cases and was unavailable in 4/91 (4.4%) due to loss of follow-up. Additionally, 13/91 (14.3%) cases could not be completely staged locoregionally by EUS due to anatomy or technical issues, such as being unable to pass the echoendoscope.

Locoregional staging in non-metastatic disease was performed in 65/91 (71.4%) cases and was accurate in 61/65 (93.8%) cases. One case of ampullary malignancy differed from the final pathologic staging (T2N0 vs. pT4N1M0) at the time of Whipple surgery two months later due to loss of follow-up; two cases differed in nodal staging on radiographic imaging (GE junction T3N1 vs. T3N2, pancreatic T2N0 vs. T2N1); and one case of rectal adenocarcinoma differed in nodal staging on operative pathology (T2N0 vs. pT2N1aM0).

Locoregional staging in metastatic disease was accurate in 15/22 (68.2%) cases. In three of the seven discrepant cases, anatomy was incompletely visualized due to a large mass or inability to pass the echoendoscope, leading to a difference in nodal staging but not tumor staging (esophageal T3Nx vs. T3N1, GE junction T3N1 vs. T3N2, rectal T4Nx vs. T4bN1). In another three discrepant cases, anatomy was sufficiently visualized but there was a difference in locoregional staging of T3 disease versus final staging of T4 disease (gastric T3N3 vs. T4N3, ampullary yT3bN1 vs. yT4N1, pancreatic T3N2 vs. T4N1). In the last discrepant case, gastric malignancy was locoregionally staged as T1aN0 vs. pathologic staging of pT2N1M1.

EUS was performed for the sampling and staging of 33 pancreatic masses. The average size was 3.7 cm (1.3–8.0). Over the study period, 30/33 (90.9%) of EUS-FNA or EUS-FNB performed for pancreatic mass yielded adequate samples for pathologic analysis; there was a trend (*p* = 0.0524) towards improved sample adequacy from the first to second data collection time period, with the predominant use of FNB. Our detection rate for malignancy for all pancreatic masses sampled via FNA or FNB was 75.8% (25/33). There was a significant increase in the detection rate from 46.2% (6/13) in the first data collection time period to 95.0% (19/20) in the second data collection time period (*p* = 0.0026).

Sensitivity was 96.2% (25/26); one case of benign EUS-FNB was confirmed to be malignant operatively. There were no cases of false positive EUS-FNA or EUS-FNB. Average follow-up of benign cases was 29.1 months (21.7–32.4). (Table 4).

EUS-FNA and EUS-FNB of pancreatic masses yielded a variety of malignancies: eleven cases of pancreatic adenocarcinoma, five cases of pancreatic neuroendocrine tumor, four cases of lymphoma, three cases of ampullary carcinoma, one case of intraductal papillary mucinous neoplasm (IPMN), and one case of metastatic small cell lung cancer.

### 3.5. Outcomes: Liver Biopsy

In the study period, there were fifteen cases of EUS-guided liver biopsy; the number of EUS-guided liver biopsies increased from four cases in the first data collection period to eleven cases in the second data collection period.

**Figure 2:**
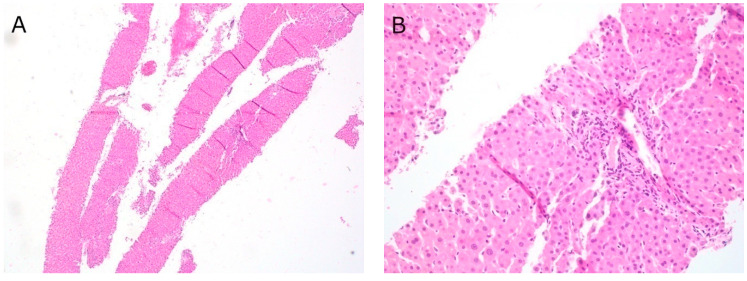
EUS-FNB liver at (**A**) 40× magnification, Hematoxylin and eosin (H&E) stain; (**B**) 200× magnification, H&E stain.

Of the fifteen cases of EUS-guided liver biopsy, specimens in three cases were obtained with both FNA and FNB needles, and specimens in eleven cases were obtained via FNB needles only; in one case needle type was not documented. A variety of needle sizes were used at the discretion of the endoscopist performing the procedure, with twelve cases obtained with a 22-G Acquire needle. There was an average of 3.6 needle passes per case, and 100% (15/15) of cases yielded adequate samples for histological analysis (Figure 2). A variety of liver pathologies were identified, including nonalcoholic steatohepatitis (NASH), autoimmune hepatitis (AIH), primary sclerosing cholangitis (PSC), and primary biliary cholangitis (PBC). There were no complications associated with any of the EUS-guided liver biopsies.

### 3.6. Outcomes: Choledocholithiasis

Evaluation for choledocholithiasis was the most common indication for EUS at our institution. Intervention was successful in 99.1% (324/327) of these cases. ERCP was deferred in 26.0% (85/327) of cases on the basis of EUS findings (Figure 1).

### 3.7. Complications

EUS-related complications occurred in three patients (0.4%); all were managed conservatively (Table 1). Two patients had superficial mucosal tears; a third patient required transfusion after EUS-FNA. There were no cases of pancreatitis or perforation. There were no cases of tumor seeding, false positive EUS-FNA or EUS-FNB, or bacteremia following EUS-FNA or EUS-FNB.

## 4. Discussion

Our study shows that the current ASGE/ACG quality metrics have all been met in a recent set of 732 consecutive EUS procedures performed at our institution for a wide variety of indications. Furthermore, some of these metrics were exceeded. Importantly, EUS changed clinical management in the majority (58.7%) of all cases. It was a well-tolerated procedure, with an EUS-specific complication rate of 0.4%.

Locoregional malignancy staging in non-metastatic disease was accurate in 93.8% of cases, while in metastatic disease it was accurate in 68.2% of cases. Other studies have documented similar rates in a variety of malignancies. EUS is highly accurate in differentiating T1-2 from T3-4 gastric cancer [11], with deficiencies primarily in the nodal staging of gastric cancers and in the depth of large tumors [12]. EUS is accurate in staging superficial esophageal cancers, including differentiating between T1a and T1b [13,14]. In rectal cancer, it is accurate for T but not N staging [15]. In pancreatic cancer, studies vary widely in the accuracy of EUS for both T and N staging, as well as for the assessment of surgical resectability [16,17,18,19,20]. Limitations in staging of metastatic disease could be due to the large size of a mass, visibility in passing an endoscope, or limitations in detecting distant disease.

Our EUS practice met ASGE/ACG targets for malignancy, with an overall detection rate of 75.8% and sensitivity of 96.2% for pancreatic masses sampled via EUS-FNA or EUS-FNB. Our institution, in which rapid on-site cytopathology is not readily available, has recently switched to the predominant use of EUS-FNB needles for tissue acquisition. This likely accounted for the increased detection rate (up to 95.0%) in the second data collection time period. Our experience correlates with published data on increased EUS-FNB yield compared to FNA, thus alleviating the need for rapid on-site cytopathology [21,22,23]. A recent meta-analysis of 11 studies has shown the superiority of FNB over FNA in the evaluation of pancreatic masses, with increased diagnostic accuracy (87% vs. 81%), cytopathologic accuracy (89% vs. 82%), and decreased mean passes required (1.6 vs. 2.3), while being equally safe [24]. The 1365 patients in these studies were sampled with a variety of FNA and FNB needle sizes and tip designs, and they yielded satisfactory results. A meta-analysis looking at needle size and type for sampling pancreatic masses evaluated 27 studies with a total of 2711 participants. They noted no difference between 25-gauge and 22-gauge FNA (RR 1.03), and also no difference between 22-gauge FNA and 22-gauge FNB (RR 1.03), with regard to diagnostic accuracy, sample adequacy, and histologic core procurement [25]. Furthermore, 16 of the studies did not have rapid on-site pathology available; they noted no difference in FNA and FNB in the absence of on-site pathology, even in more difficult to access lesions. A recent single-center, randomized trial of 150 patients found no difference between a Franseen and fork-tip needle in diagnostic accuracy (85.3% vs. 90.7%) and histologic yield (94.7% vs. 96.0%) in a variety of lesions, with a similar safety profile [26].

FNB has also emerged as an alternative to transjugular or percutaneous approaches for liver biopsy. With widespread availability of FNB needles, our institution moved from percutaneous to EUS-guided liver biopsy, resulting in an increase in the number of biopsies with 100% yield and no complications. Our data indicate that EUS-guided liver biopsy is both well tolerated and efficient. For patients undergoing evaluation for liver function test abnormalities, in a single procedure, EUS allows for not only exclusion of biliary pathology, but also liver biopsy, thus avoiding an additional procedure of percutaneous biopsy. This is in accordance with other recent studies, including a meta-analysis of nine studies and 437 patients, which has shown that EUS-guided liver biopsy has a 93.9% rate of histologic diagnosis, and a 2.3% rate of adverse events, which is comparable to transjugular or percutaneous liver biopsy [27,28].

Our experience in EUS for the evaluation of choledocholithiasis parallels other studies [29,30]. Importantly, ERCP was avoided in 26.0% of choledocholithiasis cases. Of note, at our institution, we chose to perform single-session EUS/ERCP for the evaluation of choledocholithiasis in patients with high-risk predictors, as we found EUS useful in ERCP procedural planning. Of the 31 cases (4.2%) that did not meet an ASGE indication for EUS according to the 2010 guidelines, thirty of these cases were in such patients. Ten of the thirty cases with elevated total bilirubin had normal common bile ducts upon imaging and would have warranted additional imaging according to new 2019 ASGE guidelines [7]. In these new guidelines, patients with an intermediate pre-test probability of choledocholithiasis should undergo EUS or magnetic resonance cholangiopancreatography (MRCP). A recent meta-analysis comparing EUS to MRCP evaluated five studies and a total of 272 patients; both EUS and MRCP had high specificities of 0.90 and 0.92, respectively, although EUS has a higher sensitivity than MRCP (0.97 vs. 0.87) [31]. This higher sensitivity is postulated to be due to better detection of small stones by EUS compared to MRCP, but there were not enough data to perform a statistical analysis for this hypothesis.

The main limitations of this study are its retrospective nature and being a single-center experience. Other limitations include relatively small cohort size, heterogeneity in needle type and tissue processing techniques, and the intentional shift from using FNA to FNB needles in our practice over time.

Overall, EUS has been an indispensable tool at our large tertiary safety net hospital with a diverse patient population. All ASGE and ACG target goals were met or exceeded. EUS led to a change in clinical management in the majority of cases, with a low complication rate. Implementation of EUS-FNB for the evaluation of pancreatic masses significantly increased EUS diagnostic yield. The improved quality of tissue acquisition with FNB is particularly important considering the common problem of limited availability of rapid on-site cytopathology. In addition, the data highlight the utility of EUS for sampling of liver tissue. Our study demonstrates the usefulness of monitoring quality metrics; broadening this experience may lead to further refinement of the guidelines on outcomes of endoscopic procedures.

## Figures and Tables

**Table 1:** Demographics and procedure metrics. EUS: endoscopic ultrasound; ERCP: endoscopic retrograde cholangiopancreatography; FNA: fine needle biopsy; FNB: fine needle aspiration

EUS *n* = 732	
Age (years), mean (range)	51.0 (15–92)
Gender	
Male, *n* (%)	284 (38.8%)
Female, *n* (%)	448 (61.2%)
Procedures	
Single-session ERCP	342 (46.7%)
Number of FNA/FNB performed, *n* (%)	106 (14.5%)
Procedure urgency	
Elective, *n* (%)	364 (49.7%)
Urgent, *n* (%)	349 (47.7%)
Emergent, *n* (%)	19 (2.6%)
Intervention successful, *n* (%)	715 (97.7%)
Sedation-related procedure termination, *n* (%)	1 (0.1%)
Complications	3 (0.4%)

**Table 2:** EUS performance in the study cohort against American Society for Gastrointestinal Endoscopy (ASGE) and American College of Gastroenterology (ACG) quality metrics. AJCC: American Joint Committee on Cancer

	Quality Metric	
	Study Cohort, %, (*n*)	ASGE/ACG
Pre-procedure quality metrics		
Performed for indication	95.8% (701/732)	>80%
Prophylactic antibiotics for FNA of cyst	100% (14/14)	>98%
Intra-procedure quality metrics		
Photo or video documentation	97.5% (714/732)	N/A
Documentation of sedation-related events	100% (732/732)	>98%
Documentation of appropriate structures	100% (732/732)	>98%
Staging by AJCC	100% (91/91)	>98%
Diagnostic rate of adequate sample in all solid lesion sampling	87.9% (80/91)	≥85%
Diagnostic rate for malignancy, FNA/FNB of pancreatic mass	75.8% (25/33)	≥70%
Sensitivity for malignancy, FNA/FNB of pancreatic mass	96.2% (25/26)	≥85%
Post-procedure quality metrics		
Plan for specimen follow-up	100% (106/106)	>98%

**Table 3:** Indications for EUS.

Indication	%, (*n*)
Benign	62.7% (459/732)
Stone	44.7% (327/732)
Pancreatitis	6.4% (47/732)
Biliary tree	5.2% (38/732)
Liver	2.6% (19/732)
Abscess	1.2% (9/732)
Incontinence	1.0% (7/732)
Other	1.6% (12/732)
Malignancy	37.3% (273/732)
Gastric	9.0% (66/732)
Pancreatic mass	8.3% (61/732)
Rectal	6.6% (48/732)
Pancreatic cyst	3.3% (24/732)
Esophageal	2.9% (21/732)
Biliary tree	2.9% (21/732)
Extraluminal	2.0% (15/732)
Small bowel	1.9% (14/732)
Mediastinum	0.4% (3/732)

**Table 4:** Solid pancreatic mass detection rate with EUS-FNA and EUS-FNB.

	Detection Rate, %, (*n*)	Sensitivity, %, (*n*)	Sample Adequacy, %, (*n*)
Overall	75.8% (25/33)	96.2% (25/26)	90.9% (30/33)
1st collection period predominantly FNA	46.2% (6/13)	85.7% (6/7)	76.9% (10/13)
2nd collection period predominantly FNB	95.0% (19/20)	100.0% (19/19)	100.0% (20/20)

## Data Availability

The data presented in this study are available on request from the corresponding author. The data are not publicly available due to patient privacy IRB requirement.
